# Asymmetry in inward- and outward-affinity constant of transport explain unidirectional lysine flux in *Saccharomyces cerevisiae*

**DOI:** 10.1038/srep31443

**Published:** 2016-08-23

**Authors:** Frans Bianchi, Joury S. van ‘t Klooster, Stephanie J. Ruiz, Katja Luck, Tjeerd Pols, Ina L. Urbatsch, Bert Poolman

**Affiliations:** 1 Department of Biochemistry, University of Groningen, Groningen Biomolecular Sciences and Biotechnology Institute, Nijenborgh 4, 9747 AG Groningen, The Netherlands; 2 Zernike Institute for Advanced Materials, University of Groningen, Nijenborgh 4, 9747 AG Groningen, The Netherlands; 3 Department of Cell Biology and Biochemistry, Texas Tech University Health Sciences Center, Lubbock, TX, USA

## Abstract

The import of basic amino acids in *Saccharomyces cerevisiae* has been reported to be unidirectional, which is not typical of how secondary transporters work. Since studies of energy coupling and transport kinetics are complicated *in vivo*, we purified the major lysine transporter (Lyp1) of yeast and reconstituted the protein into lipid vesicles. We show that the Michaelis constant (K_M_) of transport from out-to-in is well in the millimolar range and at least 3 to 4-orders of magnitude higher than that of transport in the opposite direction, disfavoring the efflux of solute via Lyp1. We also find that at low values of the proton motive force, the transport by Lyp1 is comparatively slow. We benchmarked the properties of eukaryotic Lyp1 to that of the prokaryotic homologue LysP and find that LysP has a similar K_M_ for transport from in-to-out and out-to-in, consistent with rapid influx and efflux. We thus explain the previously described unidirectional nature of lysine transport in *S. cerevisiae* by the extraordinary kinetics of Lyp1 and provide a mechanism and rationale for previous observations. The high asymmetry in transport together with secondary storage in the vacuole allow the cell to accumulate basic amino acids to very high levels.

Amino acids are imported into *Saccharomyces cerevisiae* by secondary active transporters that belong to the APC (amino acid/polyamine/organocation) superfamily. The APCs of yeast are driven by the proton motive force (PMF) and couple the uphill transport of amino acids to the downhill transport of protons. As expected for secondary transporters dissipation of the PMF leads to solute export down the concentration gradient, as has been shown in yeast for most amino acids^1^,^2^. However, little or no export of basic amino acids is observed in *S. cerevisiae* when the PMF is dissipated^2^,^3^. Four APC proteins are responsible for the import of basic amino acids: Lyp1 Can1, Gap1 and Alp1^4^; Lyp1 is the major transporter for lysine, Can1 for arginine. Deletion of all four genes abolishes the transport of basic amino acids across the plasma membrane (PM) of yeast^5^.

Mechanistic and bio-energetic studies of amino acid transport *in vivo* are hampered by the sequestration of solutes in the organelles, their metabolism inside the cell and the difficulty to manipulate the ion gradients. Furthermore, transcriptional or post-translational regulation of transport and removal of the transporter from the PM influences the measured activity. To study transport independent of these challenges, Can1p has been studied in hybrid plasma membrane vesicles. In this system the transport of arginine via Can1 was found to be unidirectional^6^, but this conclusion was revisited in a later study when efflux was observed under conditions where cells were growing exponentially^7^.

The consensus is that secondary transporters work via the principle of alternating access in which a solute can dock on a single binding site that is alternately accessible from either side of the membrane. Solute binding on the *cis* side triggers a conformational change and the solute is released on the *trans* side. The directionality of transport is dictated by the driving force, and for solute proton symport with a PMF inside negative and alkaline relative to the outside, there is net import of solute until the solute concentration gradient comes into equilibrium with the electrochemical proton gradient. For lysine or arginine symport with a proton, the situation is a bit more complicated as the membrane potential acts as driving force via the charge on the proton and the solute. For all transporters studied to date, thermodynamic equilibrium of the solute and electrochemical proton gradient is typically not reached because of some leak pathway^8^.

The mechanism of alternating access is supported by structural data of several secondary transporters and is perhaps best documented for the proteins with the so-called LeuT-fold. The LeuT-fold consists of two times five transmembrane segments that are related to each other by a pseudo-twofold axis^9^,^10^. Collectively, the structures of several LeuT-fold proteins show a high symmetry between their respective inward and outward-facing conformational states and strongly support the alternating access mechanism^11^,^12^.

Here, we study the mechanism of lysine transport via *S. cerevisiae* Lyp1, an APC-family protein with the LeuT-fold. We confirm previous observations that lysine transport *in vivo* is highly directional, using strains deficient in lysine breakdown and vacuolar storage. We purified and reconstituted Lyp1 in lipid vesicles, and benchmarked the energetics and kinetics of lysine transport of Lyp1 against those of a bacterial homologue, LysP from *Salmonella typhimurium*. We show that the apparent directionality of lysine transport is due to intrinsic properties of the Lyp1 transporter, related to an extreme asymmetry in the K_M_ for inward and outward transport of lysine and a particular PMF dependence of transport, that is, when compared to LysP.

## Results

### Lysine transport in *S. cerevisiae*

Yeast cells control the influx of lysine by regulating the level of Lyp1 in the plasma membrane (PM). Regulation depends on the availability of extracellular lysine and requires specific adaptor proteins, Art1 and Art2, which recognize the N-terminal tail of Lyp1 and trigger ubiquitination by Rsp5 and subsequent endocytosis^13^,^14^,^15^. In addition, an imbalance in amino acid levels, e.g. due to excessive lysine accumulation, triggers a GAAC response^16^,^17^, which affects the trafficking of amino acid permeases and their chaperones independent of the presence of the N-terminal tail. To lower the endocytic breakdown during growth and subsequent incubations we removed the N-terminal tail of Lyp1, yielding Lyp1(62-590)-YPet; all Lyp1 constructs were tagged with YPet (or GFP), a fluorescent protein that allows localization of the transporter. The *in vivo* transport activity and Michaelis constant (K_M_) for lysine of Lyp1 was the same without or with the fluorescent protein fused to the C-terminus.

**Figure 1:**
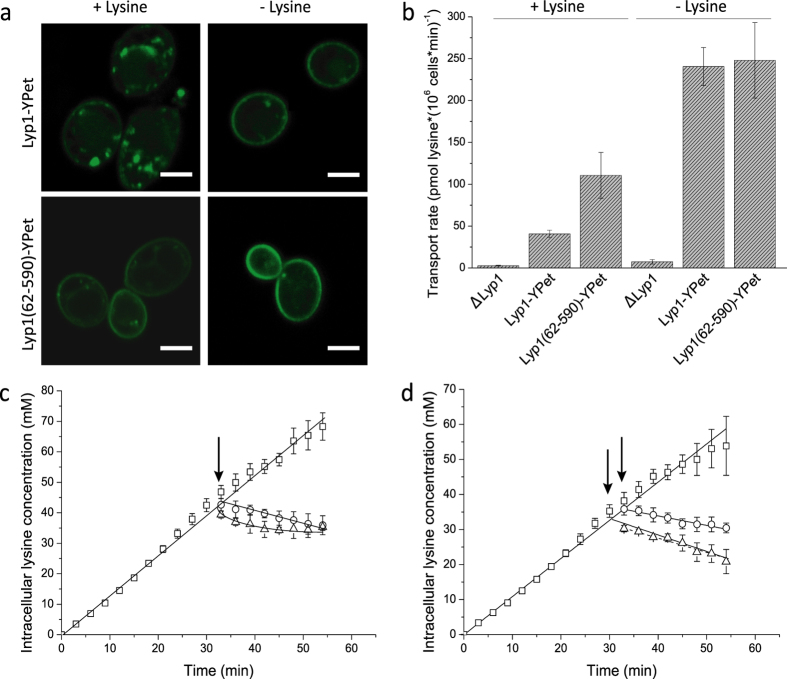
Lysine transport in *Saccharomyces cerevisiae*. (**a**) Confocal imaging of Lyp1-YPet and Lyp1(62–590)-YPet expressing cells and grown in the presence and absence of lysine. (**b**) Lysine transport rate of wild-type Lyp1 and Lyp1(62-590) expressing cells grown in the presence and absence of lysine. (**c,d**) Effect of FCCP (circles) and 100 mM lysine (500x fold excess of unlabeled solute, triangles) on the uptake of lysine into cells overexpressing Lyp1(62-590)-YPet; time-point of addition is indicated by arrows. (**c**) In *S. cerevisiae* BY4742, the initial rates of export expressed (as mM lysine*min^−1^ and SEM calculated) were 0.30 ± 0.02 and 0.28 ± 0.07 for FCCP treatment and lysine chase, respectively. (**d**) For *S. cerevisiae* BY4742 *∆vba1, ∆vba2, ∆Lys1*, the respective values were 0.25 ± 0.02 and 0.36 ± 0.04 mM lysine*min^−1^. The rates of efflux and SEM are based on biological replicates (n = 3), and the observed differences are not significant.

To minimize transcriptional regulation we expressed Lyp1 and derivatives from a multicopy plasmid under the inducible *GAL* promoter in a ∆Lyp1 strain. We compared cells expressing wildtype Lyp1-YPet and Lyp1(62-590)-YPet grown in the absence and presence of 0.5 mM of lysine. Microscopy images showed an increased level of Lyp1(62-590)-YPet in the PM as compared to wildtype Lyp1-YPet, even in the presence of external lysine (Fig. 1a). Accordingly, the rate of ^14^C-lysine import was higher in cells expressing Lyp1(62-590)-YPet (Fig. 1b). The presence of lysine in the growth medium still had an effect on the expression of Lyp1(62-590)-YPet (Fig. 1b), which may reflect the GAAC response^16^,^17^. Importantly, the mutant protein was stably maintained in the plasma membrane in subsequent transport assays. Next, we constitutively expressed Lyp1(62-590)-YPet from a multicopy plasmid under the constitutive *ADH1* promoter and verified the apparent unidirectional nature of lysine transport by adding a 500-fold excess of non-labeled lysine (exchange conditions) or by dissipating the PMF using the protonophore carbonyl cyanide-p-trifluoromethoxyphenylhydrazone (FCCP) (Fig. 1c). Only a small fraction of ^14^C-lysine was released from the cells, even upon prolonged incubation (data not shown); thin-layer chromatography confirmed that the accumulated ^14^C-lysine was not degraded (Supplementary, Fig. S1).

The apparent unidirectional transport of lysine could be partially explained by vacuolar sequestration of the amino acid. The Vba1, Vba2 and Vba3 proteins have been implicated in vacuolar transport of lysine, and deletion of the corresponding genes reduced the accumulation of lysine to ∼20% of the isogenic wildtype strain^18^. We found that Vba1 was the main contributor to vacuolar uptake of lysine, with a minor additional activity from Vba2 (Supplementary, Fig. S2 and NoteS1). We therefore deleted the *VBA1, VBA2* genes and also the *LYS1* gene to reduce the biosynthesis of lysine (*LYS1* codes for saccharopine dehydrogenase, which catalyzes the conversion of saccharopine to lysine). Also in these knockout cells, relatively little efflux of lysine was observed upon dissipation of the PMF (Fig. 1d). The rates of lysine efflux in the presence of the protonophore FCCP in *S. cerevisiae* BY4742 *∆vba1, ∆vba2, ∆Lys1* were not significantly different from those in the wildtype BY4742 strain (see also the legend to Fig. 1), and the slow efflux must thus be an intrinsic property of the transporter. The chase experiment also showed slow but significant efflux activity, indicating exchange of ^14^C-lysine with unlabeled amino acid.

The efflux rates observed in these experiments is still low given the high cytoplasmic levels of lysine, which are ∼35 mM in the *VBA* deletion strain. We next tested the possibility of regulation of transport by increased concentrations of intracellular lysine, the so-called *trans*-inhibition. In cells preloaded with up to ∼45 mM of lysine we did not observe any reduction of the initial rate of lysine transport by Lyp1(62-590)-YPet (Supplementary, Fig. S3).

### Lysine transport in proteoliposomes: yeast Lyp1 versus bacterial LysP

**Figure 2:**
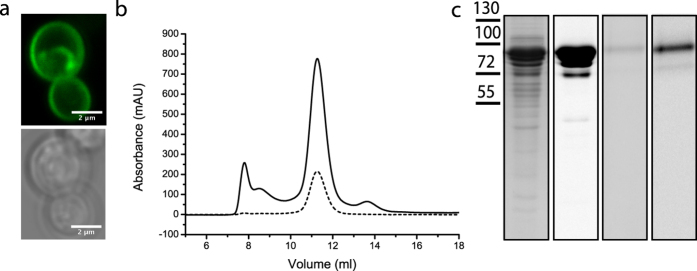
Expression and purification of Lyp1. (**a**) Confocal imaging of Lyp1-TEV-GFP-His_10_ in *P. pastoris* showing fluorescence (top panel) and bright-field image (bottom panel). Scale bar is 2 μm. (**b**) Size-Exclusion Chromatography (SEC) profiles of Lyp1-GFP after Immobilized-Metal Affinity Chromatography (IMAC); continuous and dotted lines represent absorption at 280 nm and 488 nm, respectively. (**c**) Coomassie Blue staining and in-gel fluorescence imaging of purified Lyp1-GFP (lanes 1 and 2, respectively) and Lyp1-GFP proteoliposomes (lanes 3 and 4, respectively).

To optimize the expression and to facilitate the stability screening of Lyp1, we fused GFP (Green Fluorescent Protein) to the C-terminus of Lyp1. We compared the expression of Lyp1 in *S. cerevisiae* and *Pichia pastoris*, using the *GAL* and *AOX1* promoter, respectively. We found that heterologous expression of Lyp1-GFP (*i.e*. Lyp1-TEV-GFP-his_10_) in *P. pastoris* yielded the highest protein levels, and fluorescence imaging showed that Lyp1-GFP almost exclusively localized to the plasma membrane (Fig. 2a). In parallel, we expressed a bacterial homologue of Lyp1, namely LysP from *Salmonella typhimurium* in *E. coli*, using the *pBAD* expression system as described by Kaur *et al*.^19^ (Supplementary Fig. S4). Purification of Lyp1-GFP from *P. pastoris* membranes was optimized using FSEC^20^. Lyp1-GFP and LysP were purified by sequential nickel-affinity and size-exclusion chromatography (Fig. 2b,c). Lyp1-GFP and LysP were reconstituted into lipid vesicles at molar protein-to-lipid ratio of 1 to 1000 and 1 to 800, respectively. Assuming that all protein was functionally reconstituted, vesicles with a diameter of 200 nm would on average contain two to three transporters. Since protein is lost in the reconstitution or may not be functionally incorporated into the vesicles, it is most likely that the proteoliposomes will on average have less than one functional transporter, see also our recent analysis of membrane reconstitution of another transport protein^21^.

**Figure 3:**
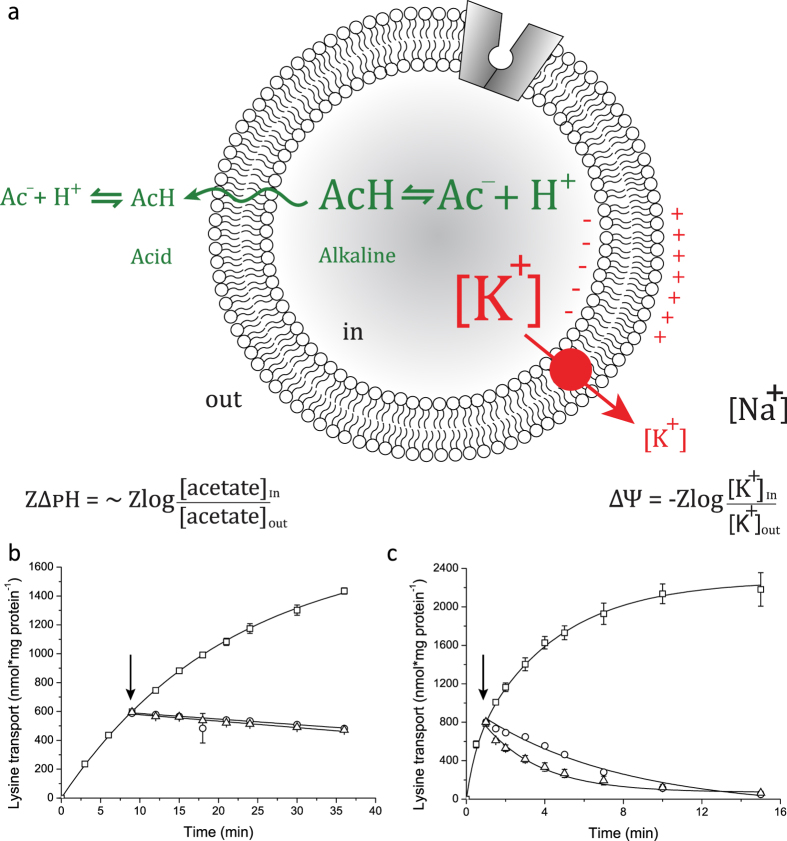
Apparent unidirectionality of Lyp1-mediated lysine transport in proteoliposomes. (**a**): Schematic showing the generation of a membrane potential (ΔΨ, red) by a valinomycin-mediated potassium diffusion potential and pH gradient (ΔpH, green) formation by an acetate diffusion potential. Together the ΔΨ and ΔpH form the proton motive force (PMF = ΔΨ−ZΔpH, where Z equals 2.3RT/F and R and F are the gas and Faraday constant, respectively, and T is the absolute temperature). (**b**) Transport of lysine by Lyp1-GFP-containing proteoliposome. (**c**) Transport of lysine by LysP-containing proteoliposomes. The effect of 10 μM FCCP (circles) and 20 mM lysine (1000x fold excess of unlabeled lysine, triangles) on the transport of 20 μM ^14^C-lysine by Lyp1 and LysP is shown; time-point of addition is indicated by arrows, and the levels of accumulation at this point are 15-fold for Lyp1 and 25-fold for LysP. All fits and errors are derived from three independent experiments.

To drive the transport of lysine into the vesicles we generated a membrane potential (ΔΨ) with and without a pH gradient (ΔpH) by diluting potassium-acetate containing proteoliposomes into sodium-phosphate with different concentrations of Na-acetate, containing the potassium ionophore valinomycin (Fig. 3a). The outward-directed potassium diffusion potential will create a ΔΨ, whose magnitude is given by the initial potassium gradient; similarly the difference in internal and external acetate concentration sets the maximal value of the ΔpH (for details see Mulligan *et al*.^22^). At a ΔΨ of −84 mV and a ZΔpH of −84 mV (external and internal pH of 6.0 and 7.3, respectively), the PMF was −168 mV, and the turnover of Lyp1 and LysP at an external lysine concentration of 20 μM was 3 and 7.5 min^−1^, respectively. Below we show how the ΔΨ and ΔpH contribute as driving force for the uptake of lysine by Lyp1 and LysP.

### Directionality and flux-force relationship of lysine transport

We allowed proteoliposomes to accumulate lysine to submillimolar levels in response to an imposed PMF of –168 mV. Lyp1- and LysP-mediated transport resulted in accumulation levels of 15–25 fold after 9 and 1 min of uptake, respectively (Fig. 3b,c), assuming a specific internal volume of 2 μl/mg of lipid^23^. We then dissipated the PMF by the addition of FCCP or chased the accumulated lysine with a 1000-fold excess of unlabeled lysine (exchange conditions). Both FCCP and the exchange conditions resulted in a rapid loss of ^14^C-lysine from the LysP-containing vesicles (Fig. 3c). On the contrary, very slow efflux of ^14^C-lysine was observed from vesicles containing Lyp1 (Fig. 3b). These experiments substantiate the findings in living yeast cells and show that Lyp1 operates highly directionally, which is unusual for a secondary transporter.

**Figure 4:**
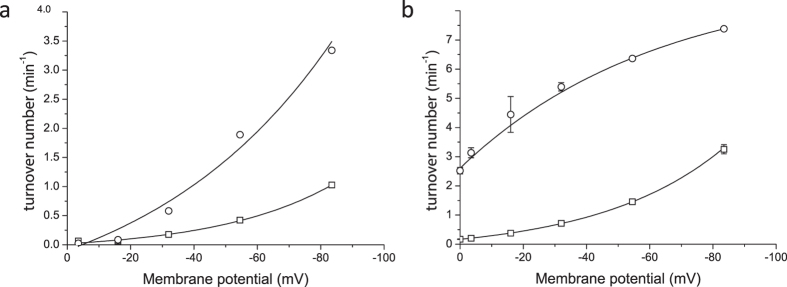
Flux-force relationships for Lyp1 and LysP. The membrane potential was varied by the potassium concentration in the external medium, and ∆Ψ was imposed without (**a**) and with (**b**) ∆pH, Z∆pH of −84 mV. Lyp1-GFP-containing proteoliposomes (squares); LysP-containing proteoliposomes (circles). Transport was assayed at 20 μM ^14^C-lysine. The turnover number is under the assumption that all protein is functionally reconstituted. All fits and errors are derived from three independent experiments.

In order to get further insights into the kinetic properties of Lyp1, we determined the initial rate of transport as a function of ΔΨ in the presence and absence of a ΔpH. Since both lysine and the proton carry a positive charge the ΔΨ contributes twice as much to the driving force as does ∆pH, assuming that lysine is transported in symport with one proton. The membrane potential was set at different values by varying the external potassium concentration, i.e. by adjusting the ratio of potassium and sodium ions to maintain isosmotic conditions. Lyp1-mediated transport increased exponentially with the membrane potential (Fig. 4a). The flux-force relationship of LysP was much steeper than that of Lyp1, and thus LysP was relatively more active at low values of the membrane potential. The ∆pH was set by varying the acetate concentration gradient, thereby varying the internal pH. The ∆pH alone supported a relatively high rate of lysine transport via LysP, which was increased about 2-fold when a ΔΨ of -84 mV was co-imposed with the ∆pH (Fig. 4b). On the contrary, below a ΔΨ of -60 mV little to no lysine influx via Lyp1 was detectable, irrespective of the presence of a ∆pH (Fig. 4a). Thus, the components of the proton motive force act very differently on the yeast and bacterial lysine transporter. The ΔΨ contributes more to lysine accumulation than the ΔpH, which is in accordance with a mechanism in which a proton is co-transported with cationic lysine.

### Kinetics of lysine transport

**Figure 5:**
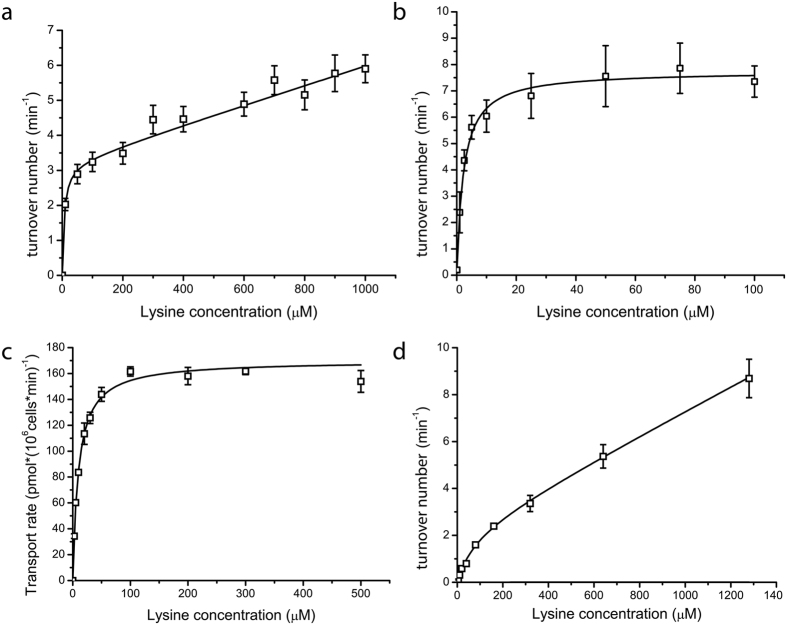
Kinetics of lysine transport. (**a**) Lysine transport of membrane reconstituted Lyp1-GFP under a PMF: the data were fitted to a single hyperbola plus a linear component (*R* factor of 0.98), (**b**)) Lysine transport of membrane reconstituted LysP under a PMF: the data were fitted to a single hyperbola (*R* factor of 0.96). (**c**) Transport of lysine by Lyp1-YPet expressed in 22∆6AAL yeast^5^ from a plasmid and under the control of the endogenous *lyp1* promoter and fit to a single hyperbola (*R* factor of 0.99). (**d**) Kinetics of lysine transport by membrane-reconstituted Lyp1-GFP under counterflow conditions. Counterflow was assayed with a an internal lysine concentration of 50 mM; higher internal concentrations of lysine could not be tested as they affected liposome formation. The data were fitted to a single hyperbola plus a linear component (*R* factor of 0.98). The rate of lysine transport (pmol*(10^6^cells*min)^−1^) (panel c) or turnover number (min^−1^) (panels a, b and d) are plotted as a function of the external lysine concentration. All fits and errors are derived from three independent experiments.

Next, we determined the Michaelis constant (K_M_) for lysine transport by Lyp1 and LysP under conditions of ΔΨ of −84 mV and ΔpH of 1.3 units (external and internal pH of 6 and 7.3, respectively; ZΔpH of −84 mV). The initial rate of uptake was determined at various concentrations of lysine (Fig. 5a,b). The data for LysP fit well to a single hyperbola (Fig. 5b) from which an apparent K_M_ of 1.5 ± 0.2 μM (SEM) was estimated. The data for Lyp1 could not be fit with a single hyperbola but instead fit well to a hyperbola plus a linear component, most likely representing high-affinity (low K_M_) and low-affinity (high K_M_) transport, respectively. The high-affinity component had a K_M_ of 20 ± 14 μM (SEM), whereas the low-affinity was not saturated at 1 mM. The high-affinity component matches well with the K_M_ of 10 ± 0.8 μM (SEM) for uptake *in vivo*, which could be fitted well to a single hyperbola (Fig. 5c) and is comparable to the K_M_ of the untagged protein^24^. Since the proteoliposomes went through multiple cycles of freezing and thawing, followed by extrusion through a polycarbonate filter (see Methods), the orientation of Lyp1 will be scrambled^25^ and the fractions of vesicles with right-side-out (RSO) and inside-out (ISO) transporters will likely be similar. The high and low K_M_ components of lysine transport observed for Lyp1 may thus correspond to the fractions of ISO and RSO reconstituted protein. These findings would thus suggest a large asymmetry in the K_M_ for out-to-in and in-to-out transport *in vivo*, which would contribute to the apparent unidirectionality of transport by Lyp1.

To substantiate the proposed asymmetry in the K_M_ for out-to-in and in-to-out transport by Lyp1, we performed so-called counterflow experiments by monitoring the influx of ^14^C-lysine in the absence of a ∆p, thereby ruling out possible effects of the membrane potential or (internal) pH on the kinetics of transport. The lysine gradient was directed outward and the degree of solute saturation of the internal site would determine the rate of transport of ^14^C-lysine. At low millimolar concentrations of lysine inside the vesicles, the rate of counterflow was very low, suggesting that the majority of RSO oriented Lyp1 molecules are not saturated with solute under these conditions (Supplementary, Fig. S5). We then determined the initial rate of uptake with 50 mM lysine inside and varied the external ^14^C-lysine concentration (Fig. 5d). Again the data fit to a single hyperbola plus linear component. We estimate the K_M_^out^ of the high-affinity component at 76 ± 28 μM (SD), which is in reasonable agreement with the value found for PMF-driven transport given the large error in estimating K_M_ values when more than one kinetic component is present. The K_M_ of the low affinity component is several orders of magnitude higher and most likely represents ISO-oriented Lyp1. Thus, in proteoliposomes with or without a PMF we observe high- and low affinity kinetics of lysine transport via Lyp1, which we interpret to reflect the RSO and ISO orientations. Accordingly, Lyp1 has a high capacity to accumulate lysine at low (<100 μM) medium concentrations, but similar or even millimolar concentrations of internal lysine are well below the K_M_ for efflux via RSO-oriented Lyp1. Translating the *in vitro* data to the *in vivo* situation, with all the Lyp1 molecules oriented RSO and at physiological (millimolar) concentrations of lysine, the efflux is limited by the high K_M_ for in-to-out transport by Lyp1, explaining the frequently reported apparent unidirectionality of lysine transport^1^,^2^.

## Discussion

The main findings of our work are summarized as follows: Basic amino acids such as lysine and arginine are accumulated by *S. cerevisiae* to relatively high levels, which relates to the large driving force for uptake because the membrane potential has twice the effect compared with the pH gradient (in case lysine is co-transported with one proton). Thus, even though Lyp1 is a relatively slow transporter, e.g. when compared to LysP, it can build up large lysine concentration gradients. In wildtype cells, part of the lysine is directed to the vacuoles through the action of Vba1 and to a lesser extent Vba2. The often described unidirectional nature of Lyp1 (and Can1)-mediated transport of lysine and arginine^6^,^7^,^26^ can be explained in the following way: (i) the Michaelis constant for in-to-out transport (K_M_^in→out^) of lysine is very high, disfavoring rapid efflux of solute even when the concentration gradient is directed outwards; and (ii) unlike LysP, Lyp1 requires a membrane potential for transport, and at low values of ΔΨ, transport is zero or very slow even in the presence of a pH gradient. Although wildtype cells store a large part of the basic amino acids in the vacuole, this storage is not the cause for the apparent unidirectionality of transport.

We also show that the slow efflux of lysine by Lyp1 is unrelated to the short life-time of the transporter in the plasma membrane as Lyp1(62–590)-YPet is stably present in the cell and still does not elicit a rapid efflux when the driving force for import is dissipated (Fig. 1a). To thoroughly characterize the kinetics of Lyp1-mediated transport, we purified and reconstituted Lyp1 in lipid vesicles composed of a mixture of yeast and *E. coli* lipids. The yeast lipids ensure that essential components from the plasma membrane of yeast are present; the *E. coli* lipids were added to form well-sealed vesicles. Since the properties of Lyp1 appeared atypical for a secondary transporter, we validated our experimental techniques by including the bacterial homologue LysP. It proved much harder to purify and reconstitute the eukaryotic Lyp1 in a functional state than it was for LysP. Critical factors for the stability and functional reconstitution of Lyp1 were low ionic strength, absence of metals, low detergent concentration and the presence of yeast lipids. Following membrane reconstitution, samples were subjected to multiple rounds of freezing in liquid nitrogen and thawing to ensure that both orientations of the protein are present in the reconstituted vesicles. We note that the calculated protein:lipid ratio would result in 2–3 transporters per vesicle, but in practice the number will be lower and most vesicles will have at most only a single functional transporter and a significant fraction will be empty. If Lyp1 exhibited strictly unidirectional behavior, the fraction of vesicles with only a single RSO Lyp1 would accumulate lysine but would not release the solute upon dissipation of the PMF or under exchange conditions. This is not what we observe; the apparent unidirectionality relates to the fact that under physiological-relevant conditions the in-to-out transport is not saturated with substrate.

**Figure 6:**
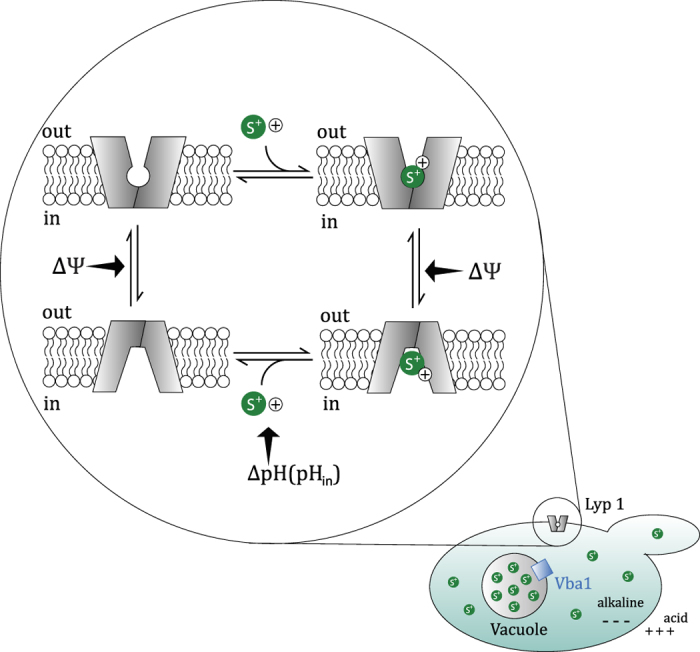
Model of mechanism of lysine transport in *S. cerevisiae.* In the cell a fraction of the lysine is accumulated in the vacuole, mostly due to Vba1-mediated uptake, which reduces the effective concentration of lysine in the cytoplasm. The in-to-out Michaelis constant (K_M_) of Lyp1 for lysine transport is very high (depicted by a different inward-facing conformation) as compared to the out-to-in K_M_ (more tight pocket for lysine, S^+^). The import rate increases with the membrane potential (ΔΨ), and most likely the ΔΨ accelerates the out-to-in or in-to-out isomerization of the protein (indicated by arrows). A pH gradient (ΔpH) increases the driving force, resulting in higher accumulation levels, but also increases the rate of transport by Lyp1. Here, the ΔpH (increased internal pH) may facilitate the release of the proton (+) when the protein is in the inward-facing conformation.

In our *in vitro* kinetic analysis we observed very different flux-force relationships for Lyp1 and LysP. The activity of LysP increases more rapidly with ΔΨ than that of Lyp1. Even more strikingly, the membrane potential dependencies become very different when the internal pH is increased from 6 to 7.3 and the ΔpH contributes additionally as driving force. The increase in internal pH may also facilitate the release of the proton when the transporter is in the inward-facing conformation (Fig. 6). The kinetics of LysP-mediated transport could in all cases be fitted with a single hyperbola, which suggests similar K_M_ values for both directions of transport (assuming that both orientations of the protein are present in the vesicles); the sensitivity and accuracy of our assays is such that a 10-fold difference in K_M_ would have been picked up. In contrast Lyp1-mediated transport required a minimum of two components to fit the data. We estimate the K_M_^in→out^ of Lyp1 to be well in the millimolar range (see Fig. S5) but could not determine the actual value because the specific activity of the radiolabel is too low at high lysine concentrations. The difference in K_M_^in→out^ and K_M_^out→in^ is not imposed by the transmembrane electrical potential or the pH gradient, as it is also observed in counterflow experiments at zero PMF. For the lactose-proton symporter LacY, the proton motive force lowers the K_M_ for import a 1000-fold (from 20–25 mM to 16 μM)^27^, while leaving the dissociation constant for galactoside binding unaffected^28^. Clearly, the low and high affinity transport of Lyp1 has a different mechanistic basis than that of LacY. *De facto* an asymmetry exists in the translocation process from in-to-out and out-to-in, and the K_M_ of Lyp1 for lysine is not dependent of the PMF. Also, the transport kinetics is distinct from that of other well-studied members of the LeuT family, including BetP, CaiT, LeuT, vSGLT and neurotransmitter transporters^29^,^30^,^31^,^32^,^33^,^34^. The asymmetry in the Michaelis constant for lysine explains the absence of efflux at ∼1 mM internal lysine in the proteoliposomes, which is well below the K_M_^in→out^ of Lyp1.

The high K_M_^in→out^ alone may not suffice to explain all the reported failures to elicit efflux *in vivo* as yeast can accumulate lysine well beyond 70 mM (based on total cell volume, including the organelle volumes). We have used the Haldane relationship^35^ to obtain an estimate of the equilibrium constant (K_eq_) of the transport reaction by using the data of Fig. 5A and assuming K_M_^in→out^ is 10 mM. In the Haldane expression Eq.1.


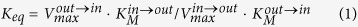


We do not know the V_max_^in→out^, but if we assume K_M_^in→out^ is 10 mM then we can estimate the V_max_^in→out^ from the data in Fig. 5A. The V_max_ would then be about 60 per min and the equilibrium constant ~50. Using this turnover number, 10^5^ Lyp1 molecules in the PM, a cytoplasmic volume of 40 fL and a free internal lysine concentration of 70 mM, it would take 2 to 2.5 hours to export half of the lysine from the cell. This is in reasonable agreement with the data presented in Fig. 1, given the uncertainty in the number of Lyp1 molecules and the fact that the turnover number *in vivo* is typically higher than in reconstituted systems with non-native lipid compositions and the fact that most likely not all protein is functionally incorporated into the vesicles^36^,^37^.

We propose a model (Fig. 6) in which the apparent unidirectional transport of lysine in *S. cerevisiae* is a consequence of: (i) asymmetry in the kinetics from in-to-out and out-to-in transport by Lyp1; and (ii) the strong dependence of the lysine flux on the driving force, resulting in very low rates of transport at low or no PMF. The secondary storage of lysine in the vacuole by Vba1-mediated uptake reduces the effective concentration of lysine in the cytoplasm but does not contribute to the directionality of transport.

In conclusion: we explain the previously described unidirectional nature of lysine in *S. cerevisiae* by the unusual kinetics of the Lyp1 transporter. The asymmetry in out-to-in and in-to-out transport may relate to two conformations of the protein, i.e. outward-open and inward-open and with high and low affinity for lysine, respectively (Fig. 6). Such a mechanism avoids leakage of the amino acid and may have physiological significance by aiding the nutrition of the cell. The mechanism of transport of lysine is in full accordance with present models of secondary transport involving alternating access, and we find no indications for additional regulation of Lyp1 that would prohibit it from exporting solute. We speculate that our findings can be translated to other well-studied transporters in *S. cerevisiae* such as Can1 and Gap1.

## Methods

### Plasmid and strain construction

The strains, plasmids and sequences of oligonucleotide primers used in this study are listed in (Supplementary Tables, 1,2 and 3) respectively. All plasmids were generated using uracil excision-based cloning. Genomic DNA isolation of *S. cerevisiae* BY4742 was carried out according to Sherman *et al*.^38^. The amplification of DNA with uracil-containing primers was performed using the polymerase PfuX7^39^. Amplified fragments were assembled into full plasmids (Table S2) by treatment with DNA glycosidase and DNA glycosylase-lyase endo VIII, commercially available as ‘USER’, following the manufacturer’s instructions (New England Biolabs, Ipswich, Ma, USA).

The plasmids pFB001, pFB004, pFB011, pFB012, pFB013, pFB014 and pFB015 were constructed by a four-way PCR fragment ligation, in which the backbone of the pDDGFP-2 vector was amplified with primer pairs Pr1/Pr2 and Pr3/Pr4. The resulting two fragments were combined with a third and fourth fragment, coding for *YPet* and the gene of interest, respectively. The fragment coding for the *YPet* gene was amplified from a synthetically generated coding sequence ordered from GeneArt (Regensburg, Germany), using primer pair Pr5/Pr6. The *LYP1* gene was amplified from *S. cerevisiae* BY4742 chromosomal DNA with primer pair: Pr7/Pr8. Ligation of the four PCR amplified fragments using USER enzyme resulted in a fusion of *lyp1* and *YPet,* separated by a sequence coding for the tobacco etch virus (TEV) protease cleavage site (GENLYFQGSGS) and followed by sequence for a His_8_ tag (i.e. *lyp1-TEV-YPet-his*_*8*_). Similar plasmids were constructed for *vba1*, *lyp1(62-590), vba1, vba2, vba2ex, vba3* and *vba3ex* in place of *lyp1*, using primer pairs Pr9/Pr10, Pr11/Pr12, Pr13/Pr14, Pr15/Pr14, Pr16/Pr17, Pr18/Pr17, respectively. In the case of *vba2ex* and *vba3ex,* template DNA was acquired from a previous study (unpublished result). For constitutive expression of *lyp1* and *lyp1(62-590)* the respective fragments were integrated into the pFB021 backbone, which was amplified using primer pair Pr1/Pr41. A restrictive digest of pDDGFP2 and pYM-N6 with *Spe*I/*Sac*I and *Sac*I/*Xba*I and ligation of the fragments coding for the backbone of pDDGFP2 and the ADH promoter of pYM-N6 resulted in the pFB021 vector. For all constructs, plasmids were isolated from the *E. coli* host and the sequences of the fusion genes were verified. For the expression of Lyp1-TEV-YPet-his in *S. cerevisiae* strain 22Δ6AAL a single copy vector was generated containing *lyp1* under its native promoter plus DNA specifying the previous C-terminally fused YPet fluorescent protein and histidine tag, separated by a TEV cleavage site. Firstly, the *lyp1* locus was cloned into pRS316 via homologous recombination, for which pRS316 was linearized by *SmaI*. The *lyp1* locus was PCR amplified, using primer pair Pr19/Pr20, and homologously recombined with the linearized backbone of pRS316 in *S. cerevisiae* BY4742. Secondly, the *lyp1* ORF of pFB017 was swapped for *lyp1-TEV-YPet-his*_*8*_ via homologous recombination, combining the PCR-amplified pFB00pre and *lyp1-TEV-YPet-his*_*8*_ ORF from pFB002, using primer pairs Pr21/Pr22 and Pr23/Pr24, respectively.

For the construction of *S. cerevisiae* BY4742 *∆ lys1, ∆vba1, ∆vba2* we made use of the *ura3* selection marker and the ability for its counter selection on 5 fluoro-orotic acid (5FOA, Thermo Fisher Scientific, Waltham, MA, USA) as described by^40^. For deletion of *vba1* and *vba2* we amplified the *ura3* cassette from pUG72 with primer pairs Pr25/Pr26 and Pr27/Pr28, respectively, which contain sequences complementary to regions upstream and downstream of the gene of interest. Similarly, to delete *lys1* from the chromosome, we amplified the *his5*-containing cassette from pUG27 using primer pair Pr29/Pr30. The amplified *vba1, vba2* and *lys1* cassettes were subsequently transformed into *S. cerevisiae* BY4742, and homologous recombination of the cassettes into the genome was selected for by growth on the corresponding depletion medium. The *ura3* marker was removed from the chromosome by recombination of its homologous flanking regions, for which we selected for growth on a medium containing 5FOA. Complete removal of the genes and the *ura3* marker was confirmed via PCR.

For the expression of LysP in *E. coli* MC1061, a pBAD expression vector was constructed similar to that by Kaur *et al*.^19^. The LysP coding fragment was acquired by amplifying STM2200 from isolated *S. typhimurium* genomic DNA, using primer pair: Pr31/Pr32. The pBADcLIC-GFP fusion vector was amplified using primer pair: Pr33/Pr34 and a two-way ligation was performed with USER; the resulting DNA was transformed into *E. coli* MC1061, which we use routinely.

For the homologous integration of Lyp1-GFP-his10 into *P. pastoris* strain SMD1163, an integrative plasmid, pSR014, was created and transformed into the host as described in the Easy Select Manual (Invitrogen, Carlsbad, CA, USA). The pSR014 plasmid was acquired by two-way ligation, using linear fragments coding for Lyp1-TEV-GFP-His_10_ and pPIC. The fragment coding for *Lyp1-TEV-GFP-His*_*10*_ was amplified with pFB020 as template and using primer pair Pr35/Pr36. The pFB020 vector is a pBADcLIC-GFP derivative that was constructed by the integration of PCR amplified *lyp1*, acquired from genomic DNA of *S. cerevisiae* BY4742, using primer pair Pr37/Pr38, and integrated into pBADcLIC-GFP using Ligation Independent Cloning (LIC) as described in Geertsma *et al*.^41^. The pPICZ fragment was amplified from the pPICZ A vector using primer pair Pr39/Pr40. The resulting plasmid pSR014 was isolated from the respective *E. coli* MC1061 strain and its sequence was verified. The plasmid was linearized using a restrictive digest with *Pme*I (New England Biolabs, Ipswich, Ma, USA) and was transformed into competent *P. pastoris* strain SMD1163, using electroporation as described in the Easy Select Manual (Invitrogen). Transformed cells were plated on increasing concentrations of YPD-zeocin (100, 500 and 1000 μg/mL). A transformed strain for expression of Lyp1-GFP-his10 was selected by testing the expression level of Lyp1-TEV-GFP-his_10_ from over 30 strains. The strain yielding the highest expression (obtained at 1000 μg/mL of zeocin) was used for Lyp1-TEV-GFP-his_10._

### Preparation of *S. cerevisiae* cells for *in vivo* transport assays and fluorescence imaging

*S. cerevisiae* cells were grown in synthetic uracil and lysine dropout medium containing; 2% (w/v) glucose or raffinose, 0.67% (w/v) yeast nitrogen base without amino acids, 2 gr/L drop-out lysine, uracil Kaiser mix. Constitutive expression of Lyp1 from pFB021 and Lyp1(62-590) from pFB022 was done in BY4742 or BY4742 *∆lys1, ∆vba1, ∆vba2* strains, which were cultivated in the presence of glucose and absence of lysine and uracil and supplemented with 800 mg/L lysine-lysine dipeptide. BY4742 or BY4742 *∆lyp1* strains carrying plasmids with a Gal promoter were grown in the presence of raffinose with 800 mg/L lysine-lysine dipeptide or 69 mg/L lysine, were induced with 0.2% (w/v) galactose for 2 hours prior to the transport assay or fluorescence imaging. For the determination of the K_m_ for Lyp1 *in vivo* Lyp1-YPet was constitutively expressed from pFB018 in the 22∆6AAL^5^ strain and cultivated in the presence of glucose and 200 mg/L lysine-lysine dipeptide and absence of lysine and uracil. Cultures were grown at 30 °C with 180 RPM shaking. Subcultures were grown for two-to-three consecutive days and never exceeded an OD_600_ of 1. Cells were centrifuged at 3,000 × g for 5 min at 4 °C, supernatant was decanted and cells were suspended in ice-cold 100 mM potassium phosphate, 10 mM glucose, pH 6.0. This step was performed twice before suspension of the cells to an OD_600_ of 5.

### Fluorescence imaging

Fluorescence live cell imaging was performed on a LSM 710 commercial scanning confocal microscope (Carl Zeiss MicroImaging, Jena, Germany), equipped with a C-Apochromat 40x/1.2 NA objective, a blue argon ion laser (488 nm) and a red He-Ne laser (633 nm). Cells were immobilized between a glass slide and coverslip. Images were obtained with the focal plane positioned at the mid-section of the cells.

### *In vivo* transport assays

Each assay contained cells at OD_600_ of 0.5. All transport assays were performed in 100 mM potassium phosphate, 10 mM glucose, pH6.0 at 30 °C and using 200 μM L-[^14^C(U)]-lysine (unless otherwise indicated). Samples were mixed by magnetic stirring. At given time intervals 50 μL or 100 μL samples were taken and quenched in 2 mL ice-cold ‘stop’ buffer of the same composition as the external buffer but without lysine. Samples were rapidly separated from external buffer and collected by filtration onto a 0.45 μm pore size nitrocellulose filter (GE-Healthcare, Little Chalfont, UK), and washed with another 2 mL of the same solution. Filters were dissolved in 2 mL of scintillation solution (Emulsifier^plus^, PerkinElmer, Waltham, MA, USA) and vortexed before radioactivity was determined by liquid scintillation counting (Tri-Carb 2800TR liquid scintillation analyzer, PerkinElmer).

Samples were normalized to 10^6^ cells by cell counting using a flow cytometer (BD Accuri™, Durham, USA) and if, the intracellular lysine concentration was calculated assuming an internal volume of ~60 fl per cell.

### Expression of Lyp1 in *P. pastoris*

For the expression of Lyp1-TEV-GFP-his_10_, hereafter and in the rest of the study called ‘Lyp1’, *P. pastoris* SMD1163-Lyp1-TEV-GFP-his_10_ cells were precultured at 30˚C in minimal media containing 2% glycerol, 1.34% yeast nitrogen base without amino acids, 0.004% L-histidine, and 0.00004% d-Biotin as described in the Easy Select Manual (Invitrogen, Carlsbad, Ca, USA). Cells were grown to an optical density at 600 nm (OD_600_) of 0.6–0.8. Cells were then diluted to an OD_600_ of 0.05 in the same minimal medium where glycerol was replaced with 2% (w/v) mannitol and cultivated at 30 °C under aeration by shaking at 180 rpm. Induction of Lyp1-GFP was achieved by the addition of 0.5% (v/v) methanol at an OD_600_ of 1–2, cultivation was continued for 24 h. Next, the cells were cooled to 4 °C and harvested by centrifugation at 7500 x g for 15 min, washed once with in (50 mM Tris-HCL pH6.7, 1 mM EDTA, 0.6M sorbitol) and suspended to a final OD_600_ of ∼100.

### Membrane preparation from *P. pastoris* cells expressing Lyp1

Cells were disrupted by three sequential passes through the T series cell disrupter (Constant Systems Ltd, Low March, Daventry, UK) at 39kpsi. After the last passage, phenylmethanesulfonylfluoride (PMSF) was added to the *cell* lysate to a concentration of 1 mM. Intact cells and large debris were removed by centrifugation at 18,000 × g for 30 min at 4 °C. The supernatant was transferred and crude microsomal membranes were isolated by ultracentrifugation at 186,000 × g for 2 h. Membranes were suspended to homogeneity using a potter Elvehjem tissue grinder in Resuspension buffer (20 mM Tris-HCl (pH7.5), 0.3M sucrose, 0.1 mM CaCl_2_, 1 mM PMSF plus one tablet of protease inhibitor (cOmplete Mini EDTA-free™, ROCHE) and 1 mM pepstatin). Aliquots of 1 mL were snap frozen in liquid nitrogen and stored at −80 °C.

### Purification of Lyp1

Lyp1 was purified from microsomal membranes which were diluted and solubilized into solubilisation-buffer: (50 mM Ammonium-acetate pH 7.5, 50 mM NaCl, 10% (v/v) glycerol, 2 mM PMSF, 15 mM Imidazole and 1% (w/w) β-D-Dodecylmaltoside) to a protein concentration of 5 mg/mL and incubated for 30 min at 4 °C with slow agitation. The insoluble pellet was removed by ultracentrifugation at 444,000 × g for 20 min. The supernatant was incubated with 0.5 mL volume of Ni-Sepharose resin under slow agitation at 4°C for 1 h. The resin was collected into a column and the Ni-Sepharose was sequentially washed with 10 column volumes of wash-buffer: ‘bufferP’: (50 mM ammonium-acetate pH 7.5 (at 24 °C), 50 mM NaCl, 10% (v/v) glycerol and 0.020% (w/v) DDM) containing 30 mM imidazole pH7.5 and with bufferP containing 50 mM L-histidine. The protein was eluted with 2.5 mL of elution-buffer: bufferP containing 235 mM L-histidine applied in steps of 0.5 mL with 10 min interval. Na-EDTA 500 mM was added to a final concentration of 5 mM protein fractions were pooled and concentrated to a volume of 0.5 mL on a Viva Spin column (Sartorius Stedim, vivaspin 500) with a 100 kDa cut off by centrifugation 18,000 × g at 4 °C. The protein sample was loaded onto a Superdex200 10/300 GL column (GE-Healthcare, Little Chalfont, UK) attached to an AKTA purifier (GE-Healthcare, Little Chalfont, UK) equilibrated with bufferP containing 100 mM L-histidine. Protein was analyzed by SDS-PAGE and the protein concentration in mg/mL was determined at 280 nm absorption on a NanoDrop ND-1000 spectrophotometer (Isogen lifescience), using the calculated extinction coefficient of 126,110 M^−1^cm^−1^ and molecular mass of 97.7 kDa as determined by ProtParam: http://web.expasy.org/protparam/. Samples were dissolved in Laemmli buffer and qualitatively analyzed for purity and resolved on 12% polyacrylamide gels by SDS-PAGE. In-gel fluorescence images were collected prior to Coomassie staining and both were imaged using a Fujifilm LAS-3000 (Fujifilm, Tokio, Japan).

### Expression of LysP in *E. coli*

For the expression of LysP, *E. coli* cells containing the pFB019 plasmid were precultured in LB-amp (1% tryptone, 0.5% yeast extract, 1% NaCl and 100 μg/mL ampicillin) at 37 °C with 180 rpm shaking to an OD_600_ of 1–1.5. Cells were then diluted into LB-amp to an OD_600_ of 0.01 and grown to an OD_600_ of 0.7, after which 0.0005% (w/v) arabinose was added to induce *lysP* expression. Cells were incubated for another 3 hours under the same conditions. Next, the cells were cooled to 4 °C and harvested by centrifugation at 7500 x g for 15 min, washed once with lysis buffer (20 mM HEPES-NaOH, 100 mM NaCl, 2 mM EDTA, 2 mM of PMSF, pH 7.5) and suspended lysis buffer to a final OD_600_ of ∼100.

### Membrane preparation from *E. coli* cells expressing LysP and purification of LysP

Membranes were prepared from *E. coli* cells expressing LysP and LysP was purified from these membranes in a similar fashion as described for Lyp1 with altered buffer compositions and minor adjustments. Cells were disrupted by two sequential passes through the T series cell disrupter at 9 kpsi and the following buffer compositions were used: Resuspension buffer (20 mM HEPES-NaOH, 100 mM NaCl, pH 7.5 and 15% (v/v) glycerol to 400 mg/mL), Solubilization-buffer (20 mM HEPES-NaOH, 100 mM NaCl, 2 mM PMSF, 1% (w/v) DDM, pH 7.5), wash-buffer (20 mM HEPES-NaOH, 300 mM NaCl, 0.025% (w/v) DDM, 40 mM Imidazole pH 7.5), elution-buffer (20 mM Mes-NaOH, 300 mM NaCl, 0.025% (w/v) DDM, 350 mM imidazole, pH 5.9), and gel filtration buffer, (20 mM MES pH 5.9, 150 mM NaCl, 0.025% (w/v) DDM). Elution was performed in steps of 0.5 mL with 2 min intervals. Protein was analyzed by SDS-PAGE and the protein concentration using a calculated extinction coefficient of 117020 M^−1^cm^−1^ and a molecular mass of 55.2 kDa as determined by ProtParam: http://web.expasy.org/protparam/. Samples were resolved on 12% polyacrylamide gels by SDS-PAGE and stained with Coomassie Blue.

### Liposome preparation

For the reconstitution of Lyp1 into lipid vesicles, liposomes were prepared from total lipid extracts of *S. cerevisiae* (Avanti polar lipids Inc, Alabaster, AL, USA) and *E. coli* (Avanti polar lipids Inc, Alabaster, AL, USA). The lipids (20 mg/mL, in chloroform) were mixed in at 1:2 ratio (total lipid extracts of *S. cerevisiae:E.coli*). Chloroform was removed by evaporation using a rotary vaporizer (rotavapor r-3 BUCHI, Flawil, Switzerland). Lipids were suspended in diethylether followed by evaporation and finally dissolved in Buffer (50mM ammonium-acetate pH 7.5 (24 °C) and 50 mM NaCl) to a concentration of 10 mg/mL. The lipid solution was homogenized by tip sonication with a Sonics Vibra Cell sonicator (Sonics & Materials Inc. Newtown, CT, USA) at 4 °C for 1 min with 5 sec pulses and a 10 sec pause between every pulse. Amplitude was set to 60%. For the membrane reconstitution of LysP, liposomes were prepared from polar *E. coli* lipids (Avanti polar lipids inc) according to the method developed by Newman *et al*.^42^ plus 25% 1,2-dioleoyl-*sn*-glycero-3-phosphocholine (DOPC). The prepared liposomes were stocked at 20 mg/mL in liquid nitrogen to prevent oxidation. The stocked liposomes were pelleted by ultracentrifugation at 444,000 × g for 20 min and re-dissolved in the reconstitution buffer; LysP (20 mM MES pH5.9, 150 mM NaCl), Lyp1 (50 mM Am/Ac pH7.5, 50 mM NaCl). For a complete buffer exchange, liposomes were snap frozen and thawed at room temperature, and the freeze-thaw cycles were repeated two times. The liposomes were homogenized by repeated extrusion through a 400 nm polycarbonate filter, using the Liposofast LF-50 (Avestin inc, Mannheim, Deutschland) with nitrogen flow. For reconstitution of the vesicles with protein, the liposomes were destabilized using Triton X-100 and titrated to a point beyond the saturation point (*R*_*sat*_) as described by^25^,^43^; the final turbidity at 540 nm was at approximately 60% of *R*_*sat*_.

### Membrane reconstitution of Lyp1 and LysP

Purified Lyp1 ((w/w) protein-to-lipid ratio of 1:1000 ratio) and LysP (ratio of 1:800) were mixed with detergent-destabilized liposomes of the appropriate lipid composition and incubated for 15 min under slow agitation at 4 °C. Bio-beads SM-200 (Biorad, Hercules, Ca, USA), 10 mg/mg lipids) were sequentially added after 15, 30 and 60 min. The final mixture was left to incubate overnight after which a final batch of bio-beads was added and incubation continued for another 2 hours. Protein containing liposomes (proteoliposomes) were separated from the Bio-beads by filtration on a column followed by ultracentrifugation 444,000 × g at 4 °C for 35 min. Proteoliposomes were suspended in 5 mM potassiumphosphate (pH 7.0) at a concentration of 10 μg protein/mL, snap frozen and stored in liquid nitrogen. In order to exchange the inside buffers, centrifugation of the proteoliposomes at 444,000 × g at 4 °C for 35 min was followed by suspension in the desired buffer, two cycles of freezing and thawing, and eleven cycles of extrusion through a 400 nm polycarbonate filter.

### *In vitro* transport assays

Each assay contained 5 μg of membrane-reconstituted protein. Depending on the sample volume taken, this corresponds to 0.5–1 μg of protein per time-point. Assays were performed at 30 °C with 20 μM L-[^14^C(U)]-lysine (PerkinElmer, Waltham, MA, USA) (unless otherwise indicated) under continues magnetic stirring. At given time intervals samples were taken, treated and analyzed as described in “*in vivo* transport assays”.

### Formation of proton gradient (ΔpH) and membrane potential (ΔΨ)

To establish ion gradients, concentrated proteoliposomes were diluted 25-fold. Ions not present in the lumen of the proteoliposomes contribute to the external concentration. The internal buffer of the proteoliposomes was 20 mM potassium phosphate, 100 mM potassium acetate, pH 6.0. To attain only a ΔpH, proteoliposomes were diluted into 120 mM sodium phosphate, pH 6.0. The ΔΨ was set by diluting proteoliposomes into buffers at varying ratios of sodium-/potassium-phosphate (NaPi/KPi) (0–20 mM) and sodium-/potassium-acetate (0–100 mM), to which 0.5 μM valinomycin was added. A ΔpH plus ΔΨ was generated by diluting proteoliposomes into buffers with various ratios of sodium-/potassium-phosphate (0–120 mM) plus valinomycin. The maximal values of ΔΨ and ΔpH were calculated from the Nernst equation. To dissipate the PMF, generated by the imposed acetate and/or potassium diffusion potentials, 10 μM FCCP was added.

### Counterflow assays

Counterflow was performed at zero PMF. For this, various concentrations of L-[4,5-^3^H(N)]-lysine (PerkinElmer, Waltham, MA, USA) plus 100 mM KPi pH6.0 were encapsulated into the lumen of the proteoliposomes. To remove the external lysine, the proteoliposomes were washed twice by ultracentrifugation 166,000 × g at 4 °C for 35 min. Supernatant was removed and proteoliposomes were suspended in 1 mL of the same buffer and lysine was replaced by N-methyl-D-glucamine to keep the osmolality constant. Counterflow was performed under conditions as described for ‘*In vitro transport assays’* by diluting the proteoliposomes 50-fold into the same buffer (100 mM KPi pH 6.0 equiosmotic by the addition of N-methyl-D-glucamine), containing 10 μM L-[^14^C(U)]-lysine and 10 μM FCCP to prevent the build-up of any PMF.

### Data analysis

The data were analyzed in Origin (OriginLab, Northampton, MA) and the rate (*v*) versus substrate concentration [S] plots were fit with Eq. 2 and 3, respectively. The best fits from an analysis of the residuals were used for plotting the traces.


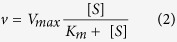



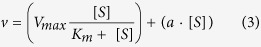


## Additional Information

**How to cite this article**: Bianchi, F. *et al*. Asymmetry in inward- and outward-affinity constant of transport explain unidirectional lysine flux in *Saccharomyces cerevisiae*. *Sci. Rep.* **6**, 31443; doi: 10.1038/srep31443 (2016).

## Supplementary Material

Supplementary Information
