# Supplementary material for: Asymmetry in inward- and outward-affinity constant of transport explain unidirectional lysine flux in Saccharomyces cerevisiae
Source: Sci Rep. 2016 Aug 23;6:31443. doi: 10.1038/srep31443 (PMC4993999; doi:10.1038/srep31443)
Supplement: Supplementary Information [file srep31443-s1.doc]

Supplementary Information

**Asymmetry in inward- and outward-affinity constant of transport explain unidirectional lysine flux in *Saccharomyces cerevisiae***

Frans Bianchi^a1^, Joury S van ‘t Klooster^a1^, Stephanie J Ruiz^a^, Katja Luck^a^, Tjeerd Pols^a^, Ina L Urbatsch^c^ and Bert Poolman^a,b,2^

^a^Department of Biochemistry, University of Groningen, Groningen Biomolecular Sciences and Biotechnology Institute, Nijenborgh 4, 9747 AG Groningen, The Netherlands

^b^Zernike Institute for Advanced Materials, University of Groningen, Nijenborgh 4, 9747 AG Groningen, The Netherlands

^c^Department of Cell Biology and Biochemistry, Texas Tech University Health Sciences Center, Lubbock, TX, USA

^1^F.B. and J.S.v.t.K. contributed equally to this work.

^2^To whom correspondence should be addressed E-mail: [b.poolman@rug.nl](mailto:b.poolman@rug.nl)

**Figure S1: Thin-layer chromatography of cell lysates after ^14^C-lysine uptake.** Phosphorescence imaging of a thin-layer chromatography plate spotted with ^14^C-lysine (1^st^ lane) or cell extracts from cells that had accumulated ^14^C-lysine; *S. cerevisiae* BY4742 (2^nd^ lane), *S. cerevisiae* BY4742 *∆vba1, ∆vba2, ∆Lys1* (3^rd^ lane).

**Figure S2:** **Sequence annotation and localization of vacuolar basic amino acid transporters.** (a) Multiple sequence alignment of Vba1, Vba2, Vba3, Vba4 and Vba5. Grey shading indicates transmembrane segments as predicted by TOPCONS ^1^. The translated upstream regions of Vba2 and Vba3 from *S. cerevisiae* S288C (isogenic to BY4742 for these genes) is included and indicated by black outlines. The upstream stop codons, which were replaced by a Leu codon to generate Vba2- and Vba3ext, are shown in red. (b) Localization of Vba1, Vba2, Vba2ext, Vba3 and Vba3ext fused to GFP and expressed in BY4742. Panels on the left show the signal from the protein (green) and from the vacuolar membrane (red, stained with FM4-64). Panels on the right show a bright-field image of the same cells. Scale bars are 2 μm.

**Note S1: Background information on vacuolar amino acid transport**

To our knowledge, only three proteins (Vba1p, Vba2p and Vba3p) have been reported to transport basic amino acids over the vacuolar membrane ^2^. Individual or combined deletions of *VBA1, VBA2* or *VBA3* were shown to reduce ATP-dependent lysine uptake by vacuolar membrane vesicles to 20–60% of the wildtype rate. Only for Vba1p was the activity and localization of the protein confirmed by gene complementation and fluorescence microscopy, using a GFP-tagged variant ^2^.

Multiple sequence alignments suggest that Vba2 and Vba3 are N-terminally truncated compared to the other members of the VBA family (Fig. S2A). Based on sequence homology, all the *S. cerevisiae* VBA proteins belong to the drug:H^+^ antiporter-2 (DHA2) family of the Major Facilitator Superfamily (MFS) ^3,4^. Members of the DHA2 family have a 14-transmembrane segment (TMS) topology, which matches the TOPCONS ^5^ predictions for Vba1, Vba4 and Vba5. Vba2 and Vba3 are predicted to only contain 12 and 11 TMS, respectively. This means that they lack TM1, 2, and/or 3 relative to the other VBAs and related MFS proteins. This is surprising given that in many other MFS proteins TM1 is implicated in gating, proton-coupling, and substrate binding ^6^.

Vba3 is a paralogue of Vba5 which arose from a genome duplication event ^7^. Analysis of the S288c genome sequence shows that Vba3 is shorter than Vba5 only because of a single base pair change (TTA to TGA, leucine -82 to STOP). Without this, the *VBA3* ORF would be upstream extended by 372 base pairs. The translated product (Vba3ext) is 99% identical to Vba5 and is also predicted to contain 14 TMS (Fig. S2A). Similarly, without a single TGA stop codon the VBA2 gene could be extended by 261 base pairs to produce a 14 TM protein (Vba2ext). The NCBI Genome database (<http://www.ncbi.nlm.nih.gov/genome/>, accessed September 2015 contains 84 *S. cerevisiae* strains that code for Vba3ext, and 86 that code for Vba2ext*.*

The annotated and extended ORFs were expressed with C-terminal YPet fusions (Fig S2B). The “short” versions resulted in intracellular aggregates and no clear membrane localization. Vba2ext-YPet localized exclusively to the vacuolar membrane, while Vba3ext-YPet was observed at the periphery of the cell and internal membranes. This is consistent with recent reports that Vba5 resides in the plasma membrane ^8^.

**Figure S3: Dependence of Lyp1 on internal lysine concentration.** (a) Initial transport rate of lysine by Lyp1(62-590)-YPet as a function of the intracellular concentration of lysine at the beginning of the experiment.

**Figure S4: Purification of LysP.** (a) Size-Exclusion Chromatography (SEC) profile of LysP after Immobilized-Metal Affinity Chromatography; the absorption at 280 nm is shown (b) Coomassie staining of purified LysP and LysP proteoliposomes analyzed by SDS-PAGE.

**Figure S5: Lysine counterflow as a function of internal substrate concentration.** Lysine counterflow at various internal concentrations of lysine: 0 (triangles), 1 mM (circles) or 10 mM (squares)’ the external concentration of ^14^C-lysine was 10 µM.

**Table S1.** *Strains used in this study*

| **Strains** | **Characteristics** | **Reference** |
| --- | --- | --- |
| *S. cerevisiae* BY4742 | *MAT*α *his3∆1 leu2∆0 lys2∆0 ura3∆0* | Brachmann 1998 et al.^9^ |
| *E. coli* MC1061 |  | Casadaban 1980 et al.^10^ |
| *S. cerevisiae* BY4742 *∆lyp1* | *MAT*α *his3∆1 leu2∆0 lys2∆0 ura3∆0 ∆lyp1::kanMx* | Giaever 2002 et al.^11^ |
| *S. cerevisiae* 22Δ6AAL | Sigma22574d  *MAT*α *gap1∆1 put4∆1 uga4∆1 ∆can1::HisG ∆lyp1::HisG ∆alp1::HisG lys2::HisG* | Fischer 2002 et al. ^12^ |
| *S. cerevisiae* BY4742 *∆lys1, ∆vba1, ∆vba2* | *MAT*α *his3∆1 leu2∆0 lys2∆0 ura3∆0 vba1∆0 vba2∆0 ∆lys1::HIS5* | This study |
| *P. pastoris* SMD1163 | Mut+, *his4, pep4, prb1* | Invitrogen (Carlsbad, CA, USA) |
| *P. pastoris* SMD1163-Lyp1-TEV-GFP-his_10_ | Mut+, *his4, pep4, prb1, Lyp1-TEV-GFP-his_10_* | This study |

**Table S2.** *Plasmids used in this study*

| **Plasmids** | **Characteristics** | **Reference** |
| --- | --- | --- |
| pDDGFP-2 | pRS426 with *gal* promoter and GFP-His fusion cassette with *ura3* selection marker | Newstead 2007 et al.^13^ |
| pYM-N6 | *Amp^R^, KanMx* chromosomal integration cassette for expression of target protein under the *ADH1* promoter | Janke 2004 et al. ^14^ |
| pBADcLIC | pBADMycHisB derivative with *MGGGFA-TEV-site-GFP-His_10_* coding region inserted in multiple cloning side | Geertsma 2007 et al. ^15^ |
| pBADcLIC-GFP | pBADMycHisB derivative with *MGGGFA-TEV-site-His_10_* coding region inserted in multiple cloning side | Geertsma 2007 et al. ^15^ |
| pRS316 | Single copy shuttle vector with *ura3* selection marker | Sikorski 1989 et al. ^16^ |
| pUG72 | *Amp^R^, Ura3* chromosomal integration cassette | Gueldener 2002 et al. ^17^ |
| pUG27 | *Amp^R^, His* chromosomal integration cassette | Gueldener 2002 et al. ^17^ |
| pSR014 | pPICZ derivative with *lyp1-TEV-GFP-his* | This study |
| pFB001 | pRS426GAL1-GFP derivative with *Lyp1* fused to *TEV-YPet-his* | This study |
| pFB004 | pRS426GAL1-GFP derivative with *vba1* fused to *TEV-YPet-his* | This study |
| pFB011 | pRS426GAL1-GFP derivative with *lyp(62-590)* fused to *TEV-YPet-his* | This study |
| pFB012 | pRS426GAL1-GFP derivative with *vba2* fused to *TEV-YPet-his* | This study |
| pFB013 | pRS426GAL1-GFP derivative with *vba2 extended* fused to *TEV-YPet-his* | This study |
| pFB014 | pRS426GAL1-GFP derivative with *vba3* fused to *TEV-YPet-his* | This study |
| pFB015 | pRS426GAL1-GFP derivative with *vba3 extended* fused to *TEV-YPet-his* | This study |
| pFB016 | pBAD derivative with *vba3* fused to *TEV-YPet-his* | This study |
| pFB017 | pRS316 derivative with *lyp1* locus | This study |
| pFB018 | pRS316 derivative with *lyp1* locus fused to TEV-YPet-his | This study |
| pFB019 | pBAD derivative with *lysp* fused to *TEV-his* | This study |
| pFB020 | pBAD derivative with *lyp1-TEV-GFP-His_10_* | This study |
| pFB021 | pRS426 with *ADH1* promoter and GFP-His fusion cassette with *ura3* selection marker | This study |
| pFB022 | pFB021 derivative *lyp1* fused to *TEV-YPet-his* | This study |
| pFB023 | pFB022 derivative *lyp(62-590)* fused to *TEV-YPet-his* | This study |

**Table S3. *Primers used in this study***

| **Primer name** | **Sequence** | **Purpose** |
| --- | --- | --- |
| Pr1 | ACCACCACCAUCATCATCATCATTAACTGCAGGAATTC | Fw primer for amplification of pDDGFP-2 vector annealing at histag for swapping c terminal fusion protein. |
| Pr2 | AGGGTAGTGCUGAAGGAAGCATACGATACCC | Fw primer for amplification of pDDGFP-2 |
| Pr3 | AGCACTACCCUTTAGCTGTTCTATATGCTGCC | Rev primer for amplification of pDDGFP-2 |
| *Pr4* | | ATTTTGGGAUCCACTAGTTCTAGAATCCGGGG | | --- | | Rev primer for pDDGFP-2 backbone amplification anneals behind *gal* promoter. |
| Pr5 | AGGGGAAAAUTTATATTTTCAAGGTTCTAAAGGTGAAGAATTATTCACTGG | Fw primer for amplification of *YPet gene* and insertion into pDDGFP-2. |
| Pr6 | ATGGTGGTGGUGGAGCTCTTTGTACAATTCATTCATACC | Rev primer for amplification of *YPet gene* and insertion into pDDGFP-2. |
| Pr7 | ATCCCAAAAUGGGCAGGTTTAGTAACATAATAACGTCC | Fw primer for amplification of *S. cerevisiae lyp1* gene for insertion into pDDGFP-2. |
| Pr8 | ATTTTCCCCUCCTGCAACAGCAGCCCAGAATTTCTC | Rev primer for amplification of *S. cerevisiae lyp1* gene for insertion into pDDGFP-2. |
| Pr9 | ATCCCAAAAUGGGACAAACACTAGACGAGACTTCAAATCTAC | Fw primer for amplification of *S. cerevisiae vba1* gene for insertion into pDDGFP-2. |
| Pr10 | ATTTTCCCCUCCAGAACTTGAACTACGTTTGTAAGTATGTTTC | Rev primer for amplification of *S. cerevisiae vba1* gene for insertion into pDDGFP-2. |
| Pr11 | ATCCCAAAAUGCATGGGTCATTGCAAGGTGG | Fw primer for amplification of *S. cerevisiae lyp1(62-590)* gene for insertion into pDDGFP-2. |
| Pr12 | ATCCCAAAAUGCATGGGTCATTGCAAGGTGG | Rev primer for amplification of *S. cerevisiae lyp1(62-590)* gene for insertion into pDDGFP-2. |
| Pr13 | ATCCCAAAAUGGAGAGTATTTCAAATTGGATCACCACTG | Fw primer for amplification of *S. cerevisiae vba2* gene for insertion into pDDGFP-2. |
| Pr14 | ATTTTCCCCUCCTCTTCTTGTTTTAGGTTTCGCCAGATTGTC | Rev primer for amplification of *S. cerevisiae vba2* gene for insertion into pDDGFP-2. |
| Pr15 | ATCCCAAAAUGGAGCTTAAATCTAGTAAACACAAAGTACTACCG | Fw primer for amplification of *S. cerevisiae vba2ex* gene for insertion into pDDGFP-2. |
| Pr16 | ATCCCAAAAUGGAGAATATGCTCATTGTCGGTAGAG | Fwprimer for amplification of *S. cerevisiae vba3* gene for insertion into pDDGFP-2. |
| Pr17 | ATTTTCCCCUCCCTTGTCTTCTAAATTATCTTCTGGTGTCTCGTC | Rev primer for amplification of *S. cerevisiae vba3* gene for insertion into pDDGFP-2. |
| Pr18 | ATCCCAAAAUGGAGGAAACTAAGTACTCTTCGCAGC | Fw primer for amplification of *S. cerevisiae vba3ex* gene for insertion into pDDGFP-2. |
| Pr19 | GGCGGCCGCTCTAGAACTAGTGGATCCCCCGATTTGAGTACTATCGCTGGC | *lyp1* allele to prs316 rev |
| Pr20 | GATAAGCTTGATATCGAATTCCTGCAGCCCATTGCCATTGGAGAAAGCCC | *lyp1* allele to prs316 fw |
| Pr21 | TCACAGAACCTCTTGCATGCC | Fw primer for amplification pFB00pre without *Lyp1* ORF |
| Pr22 | ATATATATATATATACGATGTCTTTTGTTATCGTTATAGACAATGC | Rev primer for amplification pFB00pre without *Lyp1* ORF |
| Pr23 | GCATTGTCTATAACGATAACAAAAGACATCGTATATATATATATATATGGGCAGGTTTAGTAACATAATAAC | Fw primer for amplification of lyp1-TEV-YPet-his from pFB001 |
| Pr24 | CTATTTTGAAGGCATGCAAGAGGTTCTGTGATTAATGATGATGATGATGGTGGTG | Rev primer for amplification of lyp1-TEV-YPet-his from pFB001 |
| Pr25 | CTTTGTTCTTCACTAAGCTCAGAGCCCTAGTTGTTGAGGGAACAAGAAGAAAAATCCTTCTAGGAATGAGCAATTCTATTGCATCTTTTTCAGCTCGTTTTATTTAGGTTCTATCGAGG | Fw primer for amplification of *ura3* cassette for deletion of *vba1* |
| Pr26 | GACCTTGGATTTATAAAGGTATATAATATAAAGTCTATTTTCAATTTTGTCTAGAGATCCCAATACAACAGATCAC | Fw primer for amplification of *ura3* cassette for deletion of *vba1* |
| Pr27 | GAACCGTTACAGCTTCGACGTACCAAACGATTGGTAACGAATTTAATCAGAAACTAGATATTCTACTTGACACTAAACTTTTTTTGTAAGCTCGTTTTATTTAGGTTCTATCGAGG | Fw primer for amplification of *ura3* cassette for deletion of *vba2* |
| Pr28 | CAATACTGTTAATGTATCAATTGAGTCGGTCAAGGGCATAGGTATATATTGTGTCTAGAGATCCCAATACAACAGATCAC | Fw primer for amplification of *ura3* cassette for deletion of *vba2* |
| Pr29 | CATACCATAAGATAACAACGAAAACGCTTTATTTTTCACACAACCGCAAAACGCCGGGTCACCCGGCCAGC | Fw primer for amplification of *his5* cassette for deletion of *lys1* |
| Pr30 | CAAAAAAAAATTAAACTTGTAAATGTCAGCGTAACGATAATGTATATACTTTAAATGTAAACTCGAGAGCTCGTTTAAACTGGATGGCGGCGTTAGTATCGAATCGACAG | Fw primer for amplification of *his5* cassette for deletion of *lys1* |
| Pr31 | ACCACCACCAUCATCATCATCACCATCATTAAGTCG | Fw primer for amplification of pBADcLIC backbone anneals in histag |
| Pr32 | ATGGTTAAUTCCTCCTGTTAGCCCAAAAAAC | Rev primer for amplification of pBADcLIC backbone anneals in tatabox |
| Pr33 | ATTAACCAUGGGTTCCAAAACTAAAACCACAG | Fw primer for amplification of LysP |
| Pr34 | ATGGTGGTGGUGTCCCCCTCC CTTTTTAACGCGTTCCGGGAAG | Rev primer for amplification of LysP |
| Pr35 | ATCGGTACCUAAAATGGGCAGGTTTAGTAACATAATAACGTCC | Fw primer for amplification of Lyp1-TEV-GFP-His_10_ |
| Pr36 | ATGGTGGTGGUGATGATGATGAGAACCACGACTAGTTTTGTAGAGCTCATCCATGC | Rev primer for amplification of Lyp1-TEV-GFP-His_10_ |
| Pr37 | ATGGGTGGTGGATTTGCTATGGGCAGGTTTAGTAACATAATAACGTC | Fw primer for amplification of Lyp1 for LIC |
| Pr38 | TTGGAAGTATAAATTTTCTGCAACAGCAGCCCAGAATTTCTC | Rev primer for amplification of Lyp1 for LIC |
| Pr39 | ACCACCACCAUCATCATCATTAAGTTTTAGCCTTAG | Fw primer for amplification of pPICZ |
| Pr40 | AGGTACCGAUCCGAGACGGC | Rev primer for amplification of pPICZ |
| Pr41 | attttgggauccactagttctagaGCGGCCAGCTTGGAGTTGATTG | Rev primer for amplification of pFB021 |

**References**

1. Bernsel, A., Viklund, H., Hennerdal, A. & Elofsson, A. TOPCONS: consensus prediction of membrane protein topology. *Nucleic Acids Res.* **37,** W465–8 (2009).

2. Shimazu, M., Sekito, T., Akiyama, K., Ohsumi, Y. & Kakinuma, Y. A family of basic amino acid transporters of the vacuolar membrane from Saccharomyces cerevisiae. *J. Biol. Chem.* **280,** 4851–4857 (2005).

3. Saier, M. H., Reddy, V. S., Tamang, D. G. & Västermark, A. The transporter classification database. *Nucleic Acids Res.* **42,** D251–8 (2014).

4. Reddy, V. S., Shlykov, M. A., Castillo, R., Sun, E. I. & Saier, M. H., Jr. The major facilitator superfamily (MFS) revisited. *FEBS J* **279,** 2022–2035 (2012).

5. Tsirigos, K. D., Peters, C., Shu, N., Käll, L. & Elofsson, A. The TOPCONS web server for consensus prediction of membrane protein topology and signal peptides. *Nucleic Acids Res.* **43,** W401–7 (2015).

6. Yan, N. Structural advances for the major facilitator superfamily (MFS) transporters. *Trends Biochem. Sci.* **38,** 151–159 (2013).

7. Gromadka, R., Gora, M., Zielenkiewicz, U., Slonimski, P. P. & Rytka, J. Subtelomeric duplications in Saccharomyces cerevisiae chromosomes III and XI: topology, arrangements, corrections of sequence and strain-specific polymorphism. *Yeast* **12,** 583–591 (1996).

8. Shimazu, M. *et al.* Vba5p, a novel plasma membrane protein involved in amino acid uptake and drug sensitivity in Saccharomyces cerevisiae. *Biosci. Biotechnol. Biochem.* **76,** 1993–1995 (2012).

9. Brachmann, C. B. *et al.* Designer deletion strains derived from *Saccharomyces cerevisiae* S288C: a useful set of strains and plasmids for PCR-mediated gene disruption and other applications. *Yeast* **14,** 115–132 (1998).

10. Casadaban, M. J. & Cohen, S. N. Analysis of gene control signals by DNA fusion and cloning in Escherichia coli. *Journal of Molecular Biology* **138,** 179–207 (1980).

11. Giaever, G. *et al.* Functional profiling of the *Saccharomyces cerevisiae* genome. *Nature* **418,** 387–391 (2002).

12. Fischer, W.-N. *et al.* Low and high affinity amino acid H+-cotransporters for cellular import of neutral and charged amino acids. *Plant J.* **29,** 717–731 (2002).

13. Newstead, S. *et al.* High-throughput fluorescent-based optimization of eukaryotic membrane protein overexpression and purification in Saccharomyces cerevisiae. *Proc Natl Acad Sci U S A* **104,** 13936–13941 (2007).

14. Janke, C. *et al.* A versatile toolbox for PCR-based tagging of yeast genes: new fluorescent proteins, more markers and promoter substitution cassettes. *Yeast* **21,** 947–962 (2004).

15. Geertsma, E. R. & Poolman, B. High-throughput cloning and expression in recalcitrant bacteria. *Nat. Methods* **4,** 705–707 (2007).

16. Sikorski, R. S. & Hieter, P. A system of shuttle vectors and yeast host strains designed for efficient manipulation of DNA in Saccharomyces cerevisiae. *Genetics* **122,** 19–27 (1989).

17. Gueldener, U., Heinisch, J., Koehler, G. J., Voss, D. & Hegemann, J. H. A second set of loxP marker cassettes for Cre-mediated multiple gene knockouts in budding yeast. *Nucleic Acids Res.* **30,** e23–e23 (2002).
